# Retracted: Small Molecule Kaempferol Promotes Insulin Sensitivity and Preserved Pancreatic *β*-Cell Mass in Middle-Aged Obese Diabetic Mice

**DOI:** 10.1155/2020/1013482

**Published:** 2020-09-03

**Authors:** Journal of Diabetes Research

*Journal of Diabetes Research* has retracted the article titled “Small Molecule Kaempferol Promotes Insulin Sensitivity and Preserved Pancreatic *β*-Cell Mass in Middle-Aged Obese Diabetic Mice” [1]. As raised on PubPeer [2], the article was found to contain images with signs of manipulation.

In Figures 3(a) and 3(d), there is undeclared splicing in the Glut4 rows of the Western Blots. The original images are unavailable and the authors agreed to retraction.
